# Investigating the Genetic Evolution of PCV2 and PCV3 across Six Swine Herds in China from 2020 to 2021

**DOI:** 10.1155/2023/5545806

**Published:** 2023-06-05

**Authors:** Chenchen Cui, Menghang Wang, Qianru Zhang, Shujie Wang, Hongliang Zhang, Zhijun Tian, Tongqing An, Xuehui Cai, Gang Wang

**Affiliations:** ^1^Heilongjiang Research Center for Veterinary Biopharmaceutical Technology, State Key Laboratory for Animal Disease Control and Prevention, Harbin Veterinary Research Institute, Chinese Academy of Agricultural Sciences, Harbin, China; ^2^Shandong Provincial Key Laboratory of Animal Biotechnology and Disease Control and Prevention, College of Veterinary Medicine, Shandong Agricultural University, Taian, China

## Abstract

To elucidate the prevalence, molecular evolution, and coinfection of PCV2 and PCV3 in China, we collected samples from six large-scale swine herds located in Heilongjiang, Henan, Hebei, and Hubei provinces and Xinjiang Uygur autonomous region from 2020 to 2021. Overall, 1,201 samples were analyzed and sequenced by PCR to identify the dominant genotypes of PCV2 and PCV3, and genetic variations in the capsid protein genes of PCV2 and PCV3 were evaluated. The major epidemic genotypes of PCV2 and PCV3 were different across different regions. The infection rate of PCV2 and PCV3 was 72.1% and 26.3%, respectively, and the coinfection rate was 18.6%. PCV2d was the dominant genotype across all herds. The PCV2d-positive rate in PCV2-positive samples was 93.5%–100% in five out of six herds, indicative of its predominance. PCV3 prevalence in China widely varied, and no dominant genotype was identified. Four, 21, 11, and 11 strains with PCV2a, PCV2d, PCV3a, and PCV3c genotypes were detected, respectively, with the capsid protein containing four, five, eight, and three aa sites with mutations, respectively. Overall, our findings revealed the dominant epidemic genotypes of PCV2 and PCV3 in six herds across five provinces in China, in addition to the main aa sites with mutations in the capsid protein. We believe that these results will facilitate the diagnosis, prevention, and control of PCV2 and PCV3 in China.

## 1. Introduction

Circoviruses (CVs) are the smallest nonenveloped, single-stranded, circular autonomously replicating viruses, belonging to the genus Circovirus in the family Circoviridae [1]. They contain two major open reading frames (ORF1 and ORF2), which encode a replication-associated protein (Rep) and a capsid protein on the opposite strands of the double-stranded DNA replicative intermediate [2, 3]. Four species of porcine CVs (PCVs) have been recognized: PCV1, PCV2, PCV3, and PCV4 [4–7]. PCV2 was first described in Canada in 1995 [8] and was found to cause PCV-associated diseases, including postweaning multisystemic wasting syndrome, reproductive failure, and porcine dermatitis and nephropathy syndrome (PDNS), in infected piglets [9]. PCV2 can be detected in most swine herds across the world; based on pairwise sequence comparisons and linearized phylogenetic trees, PCV2 is classified into five genotypes: PCV2a, PCV2b, PCV2c, PCV2d, and PCV2f [10–13]. PCV2b and PCV2d are the main prevalent genotypes, representing 25.8% and 60.6% of PCV2-positive clinical samples, respectively [14]. In China, PCV2d has reportedly been the predominant genotype since 2015 [14–16].

PCV3 was first detected in a swine farm in the United States in 2016; shortly afterwards, other countries and regions in Asia, Europe, and South America confirmed the presence of PCV3 in pigs [17–25]. Similar to PCV2, PCV3 has been associated with cardiac and multisystemic inflammation, PDNS, porcine respiratory disease complex, reproductive failure, and diarrhea [5, 26, 27]. A few studies have attempted *in vivo* characterization of PCV3 infection in an animal model [28, 29]. Based on the phylogenies of PCV3 complete coding sequences (ORF1 + ORF2), PCV3 can be classified into two genotypes: PCV3a and PCV3b [30].

Herein, we investigated the prevalence, molecular evolution, and coinfection of PCV2 and PCV3 in six swine herds across five provinces in China. Overall, 1,201 samples from infected pigs were evaluated; based on phylogenetic tree analysis of PCV2 and PCV3 ORF2 sequences, most of the 72.1% PCV2-positive samples were found to belong to PCV2d, and most of the 26.3% PCV3-positive samples were found to belong to PCV3a, respectively. Furthermore, 18.4% samples were coinfected with PCV2 and PCV3. These results indicate the prevalence of PCV2 despite the widespread use of PCV2 vaccines, and that further studies on PCV3-related diseases are warranted to determine the contribution of PCV3.

## 2. Methods

### 2.1. Sample Collection and Processing

From June 2020 to March 2021, 1,201 samples were collected from six large-scale swine herds located in Heilongjiang, Henan, Hebei, and Hubei provinces and Xinjiang Uygur autonomous region of China. Lymph node or lung tissues from infected pigs were assessed during the course of routine feeding. Briefly, approximately 1 g of lymph node or lung tissue was mixed with 1 mL Dulbecco's modified Eagle's medium in a 2 mL round-bottom centrifuge tube, followed by the addition of steel balls for tissue grinding. The sample was then grounded in a high-throughput grinder to a homogeneous mass. After freezing and thawing at −80°C and 22°C three times, 200 *μ*L of this processed sample homogenate was used for DNA extraction. The extracted DNA was stored at −80°C for PCV2 and PCV3 antigen detection.

### 2.2. Detection of PCV2 and PCV3

We designed specific primer pairs targeting PCV2 or PCV3 based on the entire open reading frame 2 (ORF2): PCV2-F (ATGACGTATCCAAGGAGGCGT) and PCV2-R (TTAGGGTTTAAG TGGGGGGTC) (amplicon length = 702 bp) and PCV3-F (TTAGAGAACGGACTTGTAACG) and PCV3-R (ATGAGACACAGAGCTATATTCAG) (amplicon length = 645 bp). The extracted DNA was then amplified by PCR. The 50 *μ*L reaction mixture comprised 25 *μ*L 2× Rapid Taq Master Mix (Vazyme Biotech Co., Ltd.), 3 *μ*L template DNA, 2 *μ*L of corresponding forward and reverse primers each, and 18 *μ*L deionized water. The cycling conditions were as follows: predenaturation at 94°C for 7 min, followed by 35 cycles of 94°C for 45 s, 58°C for 40 s, and 72°C for 90 s, and final extension step at 72°C for 9 min. The amplicons were then purified by E.Z.N.A.® Gel Extraction Kit, and the target band was ligated into the pMD18-T cloning vector; briefly, for ligation, 3.5 *μ*L template DNA, 1 *μ*L pMD18-T (Takara), 1 *μ*L high efficiency connecting fluid solution I, and 0.5 *μ*L water were incubated overnight at 16°C. The ligation product was transformed into competent *Escherichia coli* DH5*α* cells, and the plasmid in *E*. *coli* was extracted using the Plasmid Extraction Mini Kit (OMEGA). Subsequently, the plasmid was sequenced by Kumei Company (Jilin, China).

### 2.3. Gene Sequence Analysis

Multiple sequence alignments of PCV2/PCV3 were analyzed using ClustalW (Lynnon Co., DNAMAN software), and the maximum likelihood method in MEGA 7.0 was applied to deduce gene phylogenetic trees. PCV2 and PCV3 gene phylogenetic trees were established based on ORF2 sequences. Briefly, PCV2 phylogenetic tree was constructed based on nucleotide sequences of the capsid protein gene across different PCV2 genotypes. The Kimura 2-parameter model was chosen, which is more suitable for tree construction from nucleotide sequences. Furthermore, PCV3 phylogenetic tree was constructed based on the difference between the 24^th^ and 27^th^ amino acid (aa) sites of the capsid protein gene aa sequence, which classifies PCV3 into three clades: PCV3a, PCV3b, and PCV3c [20].

## 3. Results

### 3.1. Prevalence of PCV2

All six swine herds were positive for the PCV2 antigen. In total, 1,201 samples were collected, and 866 samples were positive for the PCV2 capsid protein antigen (infection rate = 72.1%). The antigen-positive rate was different across different swine herds (Table 1). Henan province showed the highest rate at 94.6% (106/112), and Xinjiang Uygur autonomous region of China showed the lowest rate at 30.9% (73/236).

**Figure 1:**
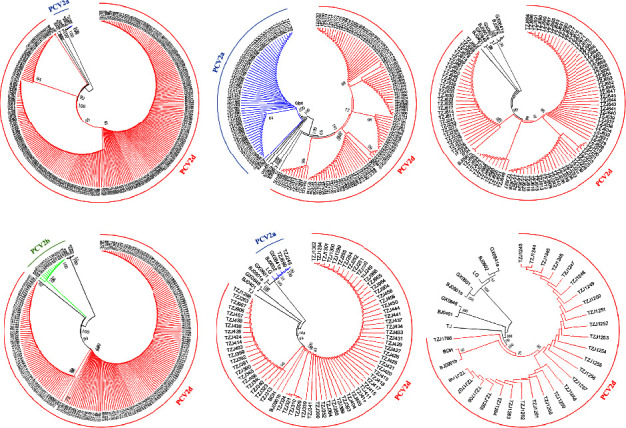
Phylogenetic analysis based on ORF2 sequences of PCV2. (a) Samples from Heilongjiang (first), (b) Heilongjiang (second), (c) Henan, (d) Hebei, (e) Xinjiang Uygur autonomous region, and (f) Hubei herds. Different colors indicate different genotypes. Blue: PCV2a; green: PCV2b; red: PCV2d. The tree was constructed by the maximum likelihood method with bootstrap values calculated for 1,000 replicates.

ORF2 analysis revealed the presence of five PCV2 genotypes in different swine herds (Figure 1). PCV2a, PCV2b, and PCV2d genotypes were detected. PCV2d was the dominant genotype and was detected in all six swine herds. The PCV2d-positive rate in PCV2-positive samples was 99.2% (262/264), 64.0% (105/164), 100% (106/106), 93.5% (217/232), 97.3% (71/73), and 100% (27/27) in Heilongjiang (two herds), Henan, Hebei, Xinjiang Uygur autonomous region, and Hubei, respectively (Figure 1). Besides, PCV2a was detected in three swine herds, and the PCV2a-positive rate in PCV2-positive samples was 0.8% (2/264), 36% (59/164), and 2.7% (2/73) in Heilongjiang (two herds) and Xinjiang Uygur autonomous region, respectively. PCV2b was only detected in Hebei province, with the PCV2b-positive rate in PCV2-positive samples being 6.5% (15/232). Among these six swine herds, one (PCV2d) or two (PCV2a and 2d or PCV2b and 2d) genotypes could coexist in the same herd; none of the samples from any herds showed the presence of >2 genotypes.

### 3.2. Analysis of aa Mutations in the Capsid Protein of PCV2 Genotype

**Figure 2:**
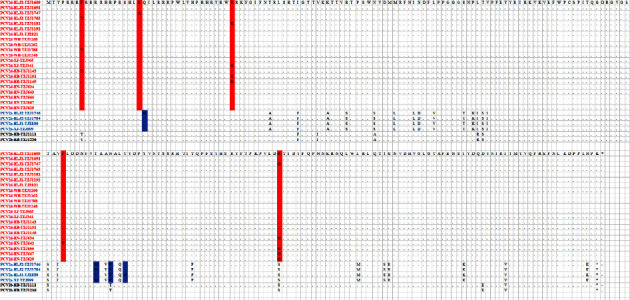
Analysis of aa mutations in the capsid protein of PCV2 genotype. 4 PCV2a, 2 PCV2b, and 21 PCV2d were selected, respectively, and their amino acid sequences were compared by using the alignment. The alignment was performed using the software MegAlign, and the major amino acid mutations are displayed in a box.

The capsid protein is the only structural protein in PCVs. The size of the capsid protein of PCV2 is 233–236 aa [31–33]. In this study, after analyzing the ORF2 sequences of all PCV2 positive samples, we successfully obtained the genome sequences of 34 PCV2 strains: four strains of PCV2 belonged to PCV2a, two strains belonged to PCV2b, and 21 strains belonged to PCV2d. The capsid protein of four strains of PCV2a was found to contain four aa sites with mutations: 21 (Q/H), 131 (M/V), 134 (P/T), and 137 (T/N). There was no difference in the aa sequence of the capsid protein between the strains with PCV2b genotype in these samples. Twenty-one strains with different PCV2d genotypes were detected, with the capsid protein containing five aa sites with mutations: 8 (F/Y), 20 (G/A), 39 (R/K), 124 (I/V), and 169 (R/G) (Figure 2).

### 3.3. Prevalence of PCV3

Of 1,201 samples, 316 were positive for the PCV3 capsid protein antigen (infection rate = 26.3%). The antigen-positive rate was different across different swine herds (Table 1). Hebei province showed the highest rate at 37.9% (102/269), and Heilongjiang (second herd) province showed the lowest rate at 15.4% (33/214).

**Figure 3:**
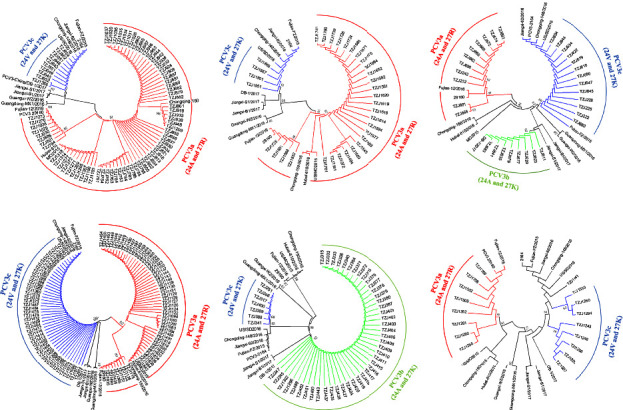
Phylogenetic analysis based on ORF2 sequences of PCV3. (a) Samples from Heilongjiang (first), (b) Heilongjiang (second), (c) Henan, (d) Hebei, (e) Xinjiang Uygur autonomous region, and (f) Hubei herds. Different colors indicate different genotypes. Red: PCV3a; green: PCV3b; blue: PCV3c. The tree was constructed by the maximum likelihood method with bootstrap values calculated for 1,000 replicates.

ORF2 analysis was performed to identify PCV3 genotypes (Figure 3). PCV3 can be divided into three clades, namely PCV3a, PCV3b, and PCV3c, based on two aa mutations (A24V and R27K) in the capsid protein [20]. Herein, we detected all three of them. PCV3c was detected in all six swine herds. As shown in Figure 3, the PCV3c-positive rate in PCV3-positive samples was 12.3% (10/81), 12.1% (4/33), 40.6% (13/32), 43.1% (44/102), 13.7% (7/51), and 52.9% (9/17) in Heilongjiang (two herds), Henan, Hebei, Xinjiang Uygur autonomous region, and Hubei, respectively. Five swine herds were PCV3a positive, with the positive rate in PCV3-positive samples being 87.7% (71/81), 87.9% (29/33), 34.4% (11/32), 56.9% (58/102), and 47.1% (8/17) in Heilongjiang (two herds), Henan, Hebei, Xinjiang Uygur autonomous region, and Hubei, respectively. PCV3b was detected in two swine herds, and the positive rate in PCV3-positive samples was 25% (8/32) and 86.3% (44/51) in Henan and Xinjiang Uygur autonomous region, respectively. Epidemiological analyses of PCV3 revealed that PCV3a was the dominant genotype across all six swine herds.

### 3.4. Analysis of aa Mutations in the Capsid Protein of PCV3 Genotype

**Figure 4:**
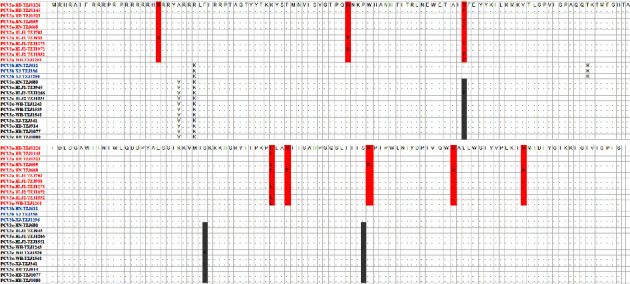
Analysis of aa mutations in the capsid protein of PCV3 genotype. 11 PCV3a, 3 PCV3b, and 11 PCV3c were selected, respectively, and their amino acid sequences were compared by using the alignment. The alignment was performed using the software MegAlign, and the major amino acid mutations are displayed in a box.

The size of the capsid protein of PCV3 is 214 aa [27]. Eleven strains with different PCV3a genotypes were detected, with the capsid protein containing eight aa sites with mutations: 20 (R/K), 56 (N/T/S), 77 (S/T), 150 (I/L), 153 (G/E), 168 (R/K), 183 (G/A), and 196 (G/A). The three strains with PCV3b genotype showed no difference in the capsid protein aa sequence, as indicated by sequencing. Furthermore, sequencing identified 11 strains with different PCV3c genotypes, with the capsid protein containing three aa sites with mutations: 77 (S/T), 137 (S/F), and 167 (S/T) (Figure 4).

### 3.5. Coinfection of PCV2 and PCV3

All six herds showed the presence of PCV2 and PCV3 in the same sample, and the coinfection rate was 23.9% (76/318), 11.7% (25/214), 25.9% (29/112), 22.3% (60/269), 8.9% (21/236), and 23.1% (12/52) in Heilongjiang (two herds), Henan, Hebei, Xinjiang Uygur autonomous region, and Hubei, respectively (Table 1).

## 4. Discussion

PCV is the smallest known DNA virus. PCV-related infections have been reported to cause serious economic losses to the domestic pig industry. PCV2 has been associated with diverse disease syndromes in pigs, such as PDNS, respiratory diseases, systemic inflammatory diseases, reproductive failure, and multisystem failure syndrome after weaning [34].

PCV2 seriously harms the health of pigs and causes huge economic losses to the pig industry in China [35, 36]. According to a retrospective study, PCV2 genotypes have undergone two important changes: in around 2003, PCV2a evolved to PCV2b, and in around 2013, PCV2b evolved to PCV2d owing to the high mutation rate of PCV2 nucleotide sequences [37, 38]. As per sequence alignment and phylogenetic analyses proposed by the European Union in 2008, PCV2 was classified into three clades: PCV2a, PCV2b, and PCV2c [12]; subsequently, in 2010, PCV2d was identified in swine herds in China [39], eventually becoming the dominant genotype across the world [24]. The new standard for PCV2 genotype update and proposal for a new genotyping methodology were reported in 2018; the genotype definition was limited to the following criteria: maximum intragenotype p-distance of 13% (calculated on the ORF2 gene), minimum cluster internal node bootstrap support of >70%, and at least 15 identified sequences [40]. Based on the new standard, PCV2b and PCV2d were the most prevalent genotypes (25.8% and 60.6%, respectively) in clinical samples from swine herds in Northeast China from 2015 to 2018 [14], indicating the predominance of PCV2d in China [14]. In this study, PCV2 infection rate was found to be 72.1%, which was higher than previously reported in the Southwest of China and in central China [15, 16], and this suggests that PCV2 is still a major pathogen to affect the health of swine herds during the course of routine feeding. Besides, of 866 PCV2-positive samples, 7.3% (63 of 866) of isolated PCV2 strains belonged to genotype PCV2a, 1.7% (15 of 866) were classified under genotype PCV2b, and 91% (788 of 866) under genotype PCV2d, which including the PCV2d-positive rate in PCV2-positive samples ranged from 93.5% to 100% in five out of six herds. This finding indicated that PCV2d is still the most dominant genotype in China.

Capsid protein is encoded by ORF2, which is involved in viral replication and host immune responses. In this study, the capsid protein of PCV2a genotypes was found to contain four aa sites with mutations: 21 (Q/H), 131 (M/V), 134 (P/T), and 137 (T/N); capsid protein of PCV2d genotypes contains five aa sites with mutations: 8 (F/Y), 20 (G/A), 39 (R/K), 124 (I/V), and 169 (R/G). These mutations locate within the second B cell-defined immunogenic surface epitope (residues 113–139) of the nucleocapsid protein [41]. Previous studies showed that a nucleocapsid surface moiety on or within epitope two is an important determinant of PCV2 virulence [42]. These mutations on capsid protein in our study, are similar to reported from other regions of China recently [15, 16], may lead to antigenic variation, and are conducive to immune escape, virulence, and transmission of PCV2 strains among vaccinated swine herds. Previous study indicates that PCV2-infected pigs and pigs with PDNS showed strong antibody responses against capsid protein oligopeptide (169–180) [43]. Whether aa 169 mutated in some of the strains as well as the other amino acid mutations at these sites in this study contribute to the immune escape of PCV2 in vaccinated swine herds warrant to further study.

PCV3 was first reported in 2016 and was detected in the United States from a sow herd, with the infection characterized by clinical symptoms of PDNS and reproductive disorders [5]. Since then, infections caused by PCV3 have been reported in swine herds in America, Asia, and Europe. The outcome varies from inapparent to severe disease (e.g., respiratory and enteric disease, multisystemic inflammation, and reproductive failure). The positive rate of PCV3 was found to be 34.7%–59.5% in samples collected from different regions in China [44]. In this study, based on 1,201 samples from six herds across five provinces, the PCV3 infection rate was 21.4%–37.9%, indicating that the prevalence and incidence of PCV3 differ in diverse breeding groups across different provinces in China. Furthermore, the epidemic of PCV3 in China showed substantial variations across various regions, implying that PCV3 continues to evolve in swine herds in China. Of 316 PCV3-positive samples, the proportion of PCV3a, PCV3b, and PCV3c was 56, 16.5, and 27.5%, respectively. PCV3a accounted for the majority, which was similar to the results reported from the Southwest of China last year [16] but was inconsistent with the findings from previous years [45, 46]. These suggest that the PCV3 still continues to spread in the swine herds of China in recent years. In this study, 11 aa sites with mutations in the capsid protein of PCV3 were detected, but whether these mutations can affect the immunogenicity of PCV3 is unclear and needs further study.

The coinfection rate of PCV3 and PCV2 in the same sample was 18.6%. The coinfection rate has been rising rapidly in China [16, 46]. Further studies are warranted to determine whether both PCV3 and PCV2 contribute to disease onset.

In conclusion, after data statistics, we found that PCV2d genotypic strain still be the most dominant strain in swine herds of China, companying with the most five aa mutation sites of capsid protein appeared in the course of massive infection. Meanwhile, since the capsid protein in PCV2a genotype has produced four aa mutation sites, we should pay close attention to PCV2a evolution under the immune pressure of a large number of commercial PCV2a genotypic vaccines. Although there is no dominant genotype in PCV3 at present, the capsid protein in PCV3a genotype contains up to 8 aa mutation sites deserves our attention. Finally, the effect of aa mutation of structural protein capsid on the pathogenicity and immunogenicity of PCV still needs further study.

## Figures and Tables

**Table 1:** Temporal prevalence rates of PCV3 and PCV2 at sample level from swine specimens collected in six swine farms of 5 provinces in China during 2020-2021.

	Sample size	PCV2 positive	%	PCV3 positive	%	PCV2/3 positive	%
Total	1201	866	72.1	316	26.3	233	19.4
Province							
Heilongjiang1	318	264	83	81	25.5	76	23.9
Heilongjiang2	214	164	76.6	33	15.4	25	11.7
Henan	112	106	94.6	32	28.6	29	25.9
Heibei	269	232	86.2	102	37.9	60	22.3
Xinjiang	236	73	30.9	51	21.6	21	8.9
Hubei	52	27	51.9	17	32.7	12	23.1

## Data Availability

The data used to support the findings of this study are included within the article.
